# Sarcoma Family Kinase-Dependent Pannexin-1 Activation after Cortical Spreading Depression Is Mediated by NR2A-Containing Receptors

**DOI:** 10.3390/ijms21041269

**Published:** 2020-02-13

**Authors:** Fan Bu, Lingdi Nie, John P Quinn, Minyan Wang

**Affiliations:** 1 Department of Biological Sciences, Centre for Neuroscience, Xi’an Jiaotong-Liverpool University (XJTLU), Suzhou 215123, China; Fan.Bu@uth.tmc.edu (F.B.);; 2 Department of Molecular and Clinical Pharmacology, Institute of Translational Medicine, University of Liverpool, Liverpool, L69 7ZB, UK; jquinn@liverpool.ac.uk

**Keywords:** cortical spreading depression, migraine, pannexin-1, sarcoma family kinases, NR2A

## Abstract

Cortical spreading depression (CSD) is a propagating wave of depolarization followed by depression of cortical activity. CSD triggers neuroinflammation via the pannexin-1 (Panx1) channel opening, which may eventually cause migraine headaches. However, the regulatory mechanism of Panx1 is unknown. This study investigates whether sarcoma family kinases (SFK) are involved in transmitting CSD-induced Panx1 activation, which is mediated by the NR2A-containing N-methyl-D-aspartate receptor. CSD was induced by topical application of K^+^ to cerebral cortices of rats and mouse brain slices. SFK inhibitor, PP2, or NR2A–receptor antagonist, NVP–AAM077, was perfused into contralateral cerebral ventricles (i.c.v.) of rats prior to CSD induction. Co-immunoprecipitation and Western blot were used for detecting protein interactions, and histofluorescence for addressing Panx1 activation. The results demonstrated that PP2 attenuated CSD-induced Panx1 activation in rat ipsilateral cortices. Cortical susceptibility to CSD was reduced by PP2 in rats and by TAT-Panx308 that disrupts SFK–Panx1 interaction in mouse brain slices. Furthermore, CSD promoted activated SFK coupling with Panx1 in rat ipsilateral cortices. Moreover, inhibition of NR2A by NVP–AAM077 reduced elevation of ipsilateral SFK–Panx1 interaction, Panx1 activation induced by CSD and cortical susceptibility to CSD in rats. These data suggest NR2A-regulated, SFK-dependent Panx1 activity plays an important role in migraine aura pathogenesis.

## 1. Introduction

Migraine is characterized by severe, episodic, and unilateral headache, which affects an estimated 12% of the population and is the second most disabling condition worldwide [1]. Migraine correlates with dysfunctions of the central nervous system and activation of the trigeminovascular system. One of the key pathophysiological basis of migraine with aura is cortical spreading depression (CSD) [2], a state of temporary excitation of neurons and glial cells followed by depression that propagates slowly across the cerebral cortex [3,4]. CSD causes a sudden rise in extracellular K^+^, nitric oxide, calcitonin gene-related peptide (CGRP) and arachidonic acid [5,6]. This phenomenon is also the likely trigger for the activation of meningeal nociceptors [7,8] and central trigeminovascular neurons [9]. Notably, a recent study demonstrates that CSD causes migraine-like headaches by triggering the neuronal pannexin-1 (Panx1) mega channel opening, leading to potentiation of neuroinflammatory responses and sustained activation of trigeminal afferents [10]. However, the molecular mechanism underlying CSD-induced Panx1 activation still remains unclear. 

Panx1 channels are large-pore ion channels that mediate ionic currents and release small molecules, such as ATP [11]. The channel activity can be regulated by activation of N-methyl-D-aspartate receptors (NMDAR) [12] and P2X7 receptors (P2X7R) [13] and high intracellular Ca^2+^ [14] via activating the inflammasome in neurons and astrocytes [15]. Upon P2X7R activation, Panx1 forms a large pore by associating with P2X7R, mediating apoptotic cell death [13], caspase-1 activation, and IL-1β release in mouse macrophage cells [16]. Moreover, the Panx1 channel is known to be regulated by sarcoma family kinases (SFK) in response to P2X7R and NMDAR activation in vitro. In cells, inhibition of SFK similarly prevents activation of the Panx1 channel upon P2X7R and NMDAR activation, whereby SFK is activated [17,18]; whilst exogenous peptides targeting the SFK-related domains of both P2X7R and Panx1 block the Panx1 channel opening [16]. It was subsequently demonstrated that NMDAR couples activated SFK to Panx1, and inhibition of SFK attenuates phosphorylation of Panx1 at amino acid tyrosine 308, induced by NMDAR activation during excitotoxicity [19]. Therefore, SFK may serve as an intermediate molecule in the signal transduction pathway resulting in the Panx1 opening, possibly in the context of migraines. 

Notably, we recently reported that SFK activation regulates CSD propagation, which then promotes SFK phosphorylation in rats [20]. A functional interaction is known to exist between SFK activity and NR2A-containing NMDAR in regulating CSD in the mouse brain slice [20]. SFK activity also regulates the phosphorylation of NR2B-containing NMDAR with subsequent central sensitization in a chronic migraine rat model [21]. Taken together and given that activation of NMDAR, mainly NR2A- and NR2B-containing receptors, play a pivotal role in CSD genesis and propagation [22,23,24], we have hypothesized that CSD-induced Panx1 activity may be SFK-dependent and this process may be regulated by NMDAR. In this report, we investigate whether the inhibition of SFK could reduce CSD-promoted Panx1 activity in rats. We next explore if the CSD-induced Panx1 activity would be dependent on SFK activity by investigating if CSD would enhance the interaction of Panx1 with phosphorylated SFK and also if disruption of SFK/Panx1 interaction would reduce CSD in mouse brain slices. We subsequently examine whether the phosphorylated-SFK and Panx1 coupling and the Panx1 activity induced by CSD could be regulated by NR2A in rats. We reason that CSD induces Panx1 activation dependent on SFK, which is mediated, at least in part, by the activation of NR2A-containing receptors.

## 2. Results

### 2.1. SFK Inhibition Reduced CSD-Induced Ipsilateral Cortical Neuronal PI Influx 

**Figure 1:**
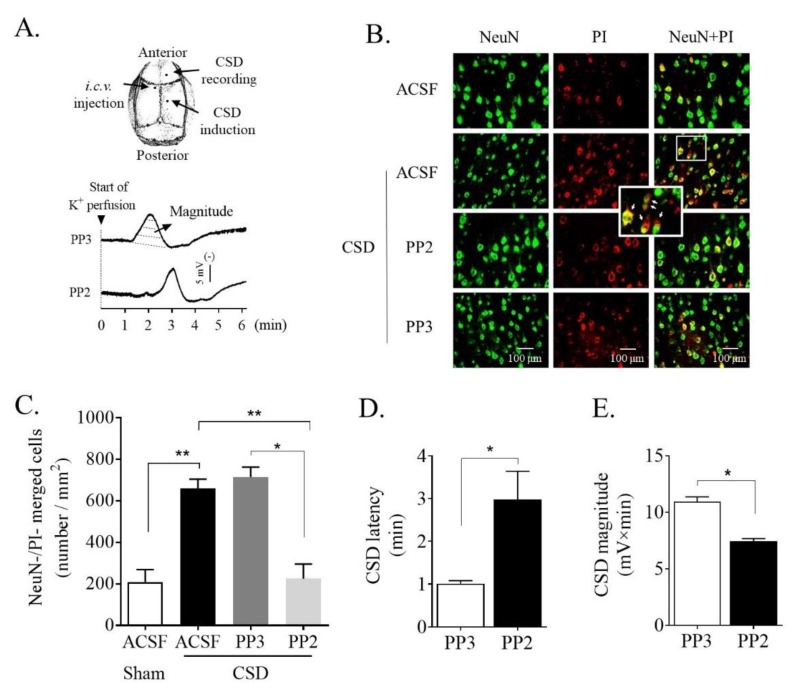
PP2, the SFK selective inhibitor, perfused into contralateral i.c.v. attenuated CSD-induced neuronal PI staining in the ipsilateral cortex of rat and reduced cortical susceptibility to CSD. (**A**) Schematic drawing shows cranial preparation and representative traces showing CSD propagation wave after i.c.v. perfusion of 2.5 nmol PP2 or its negative analog PP3. CSD magnitude (mV × minute, dashed area) and latency (L, minute) were used for quantifying CSD. (**B**) Representative images of CSD-induced PI staining (red) of NeuN positive cells (green) in layers V and VI of the sensorimotor cortices treated with ACSF, 2.5 nmol PP3 or PP2 in the absence or presence of 3M K^+^-induced CSD. PI and NeuN positive cells (yellow) indicated by arrows shown in the inset indicated increased Panx1 activity. (**C**) Effects of PP2 (*n* = 4) or PP3 (*n* = 4) on PI staining indicated by the number of merged cells (cells/mm^2^). CSD group (*n* = 5) and sham group (*n* = 6) were used as controls. (**D**,**E**) Effects of PP2 (*n* = 4) and PP3 (*n* = 4) on CSD latency and magnitude. Kruskal–Wallis test for comparison among multiple groups, followed by one-tailed Mann–Whitney test for comparisons between 2 independent groups. All the values shown are means ± SEM. Significant differences were set when * *p* < 0.05 or ** *p* < 0.01.

We examined whether SFK deactivation could reduce neuronal propidium iodide (PI) staining induced by CSD in the ipsilateral cortices of rats. In the sham group, PI-positive neurons were 210 ± 59.0 per mm^2^ in the sensorimotor cortices (*n* = 6, Figure 1B,C). This number markedly increased to 660.9 ± 43.9 per mm^2^ (*n* = 5) after CSD induction, which was significant when compared with the sham group (*p* = 0.002, Figure 1C). In the PP3 group, PI-positive neurons were 714.7 ± 48.5 per mm^2^ (*n* = 4, Figure 1C), which was not significantly different from that of the CSD group, suggesting that PP3 does not affect CSD-induced PI staining. By contrast to PP3, 2.5 nmol SFK inhibitor PP2 perfused into the contralateral i.c.v. caused a significant decrease in the number of PI-positive neurons to 225.9 ± 69.4 per mm^2^ (*n* = 4) when compared with that of the CSD (*p* = 0.008) and the PP3 group (*p* = 0.014) (Figure 1C), consistent with SFK deactivation reducing CSD-induced PI staining. Consistently, PP2 markedly prolonged CSD latency (1.01 ± 0.07 min in the PP3 group *vs.* 2.98 ± 0.66 min in the PP2 group, *n* = 4, *p* = 0.014, Figure 1D) and significantly attenuated CSD magnitude (10.94 ± 0.43 mV × minute in PP3 group *vs.* 7.43 ± 0.25 mV × minute in PP2 group, *n* = 4, *p* = 0.014, Figure 1E) compared with PP3 group. These data are in line with what was observed previously [20], suggesting that the validity of the in vivo CSD model and the associated PI staining. 

### 2.2. Blockade of SFK-Activated Panx1 Reduced Cortical Susceptibility to CSD

**Figure 2:**
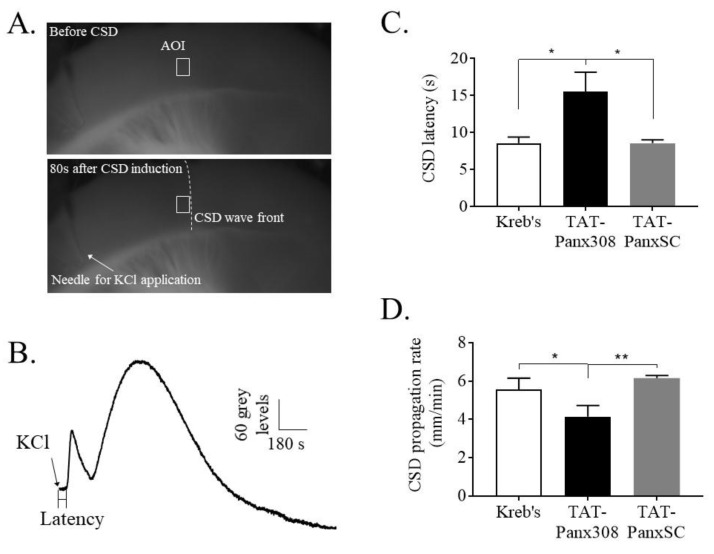
TAT–Panx308 prolonged CSD latency and reduced CSD propagation rate in the mouse brain slice. (**A**) Mouse brain slice before (upper) and after (lower) CSD induction by 260 mM KCl. An area of interest (AOI, indicated by the white rectangle) along the CSD wave front (indicated by the white dash line) was selected and was kept the same for all images of the sequence. (**B**) A representative trace of CSD wave recorded by intrinsic optical imaging on mouse brain slice. The CSD wave in a biphasic pattern was generated by plotting averaged grey level within the AOI against time. CSD latency (seconds) was the time interval from the start point of KCl application to the elicitation of depolarization. Propagation rate of CSD (mm/minute) reflects the speed by which CSD wave propagates across the cortex. (**C**,**D**) Effects of Kreb’s solution (*n* = 7), 5 μM TAT–Panx308 (*n* = 8) and 5 μM TAT–PanxSC, the negative control peptide (*n* = 8) on CSD latency and propagation rate. All the values are shown as means ± SEM. One-tailed, Mann–Whitney test was used for comparison between two independent groups. Insignificant difference was not indicated, whereas significant differences were set when * *p* < 0.05 and ** *p* < 0.01.

Since SFK inhibition was able to reduce Panx1 activation induced by CSD, we addressed whether the Panx1 activation of SFK may be essential for CSD propagation by using TAT–Panx308 that blocks tyrosine 308 of Panx1, which is the target of SFK. Each KCl application at 260 mM on the mouse brain slice led to changes in the optical signals, as indicated as a dark gray wave front slowly propagating from the KCl application site across the cortex (lower image, Figure 2A). The resulting changes in the optical signal indicate the observable CSD wave and that the pattern was biphasic (Figure 2B), which is consistent with that observed previously [20]. The data showed that pretreatment of the mouse brain slice with the TAT–Panx308 (*n* = 8) markedly prolonged the latency of CSD to 15.4 ± 7.5 s (s), compared with the scrambled control TAT–PanxSC group (8.5 ± 1.3 s, *n* = 8, *p* = 0.026) and Kreb’s control group (8.5 ± 2.2 s, *n* = 7, *p* = 0.012) (Figure 2C). Conversely, the propagation rate of CSD was significantly reduced to 4.1 ± 0.6 mm/minute by TAT–Panx308 compared with the scrambled control (6.2 ± 0.1 mm/minute, *p* = 0.002) and Kreb’s control (5.6 ± 0.6 mm/minute, *p* = 0.02) (Figure 2D).

### 2.3. CSD Promoted Ipsilateral Cortical SFK and Panx1 Interaction

**Figure 3:**
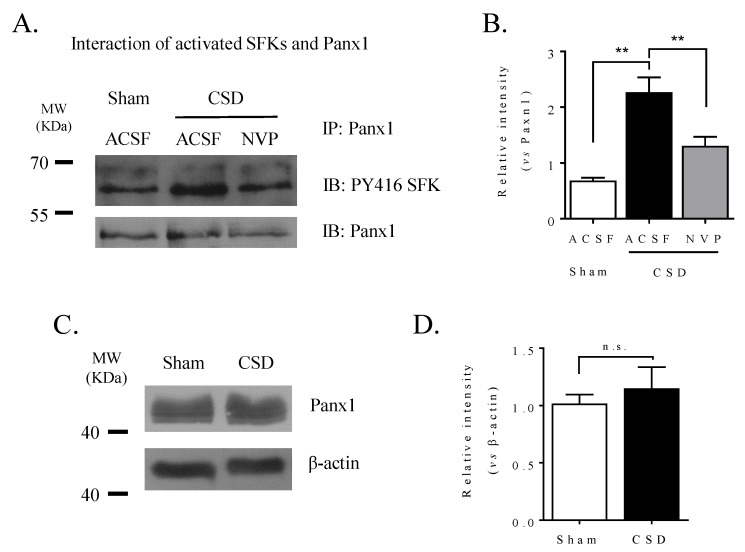
CSD promoted the interaction of activated SFK with Panx1 channels in the ipsilateral cortex of rat, which was reduced by NVP perfused into the contralateral i.c.v.. (**A**) Representative images showing co-immunoprecipitation followed by Western blot treated with ACSF or NVP (0.3 nmol) in the absence or presence of 3 M K^+^-induced CSD. Panx1 was immunoprecipitated (IP) and the pulldown of Panx1 with PY416 SFK was assessed by immunoblotting (IB). Equal loading was indicated by total Panx1 immunoblotting. (**B**) Quantitative analysis of the IB relative band intensity of PY416 SFK pulled down with Panx1 during IP in cortex treated with ACSF or NVP. *n* = 5, 6, 4 in the sham, CSD, and NVP+CSD group, respectively. Data were presented as the relative band intensity of PY416 SFK normalized to that of Panx1. (**C**) Representative images showing immunoblotting of Panx1 with or without CSD induction. (**D**) Quantitative analysis of the IB relative band intensity of Panx1 treated with ACSF or KCl. *n* = 3 in the sham and CSD group, respectively. Data were presented as the relative band intensity of Panx1 normalized to that of β-actin. One-tailed, Mann–Whitney test was used for comparisons between sham and CSD group, CSD and NVP group, respectively. All the values shown are means ± SEM. No significant difference was shown as n.s., whereas significant differences were set when * *p* < 0.05 and ** *p* < 0.01.

We previously reported that CSD promoted SFK phosphorylation in the ipsilateral cortex of rat [20]. Here we investigated whether CSD could promote the coupling of activated SFK to Panx1. Co-immunoprecipitation with the anti-Panx1 antibody followed by Western blot with the anti-PY416 SFK antibody recognized proteins at the size of 60 KDa (Figure 3A) in all the 3 groups, indicating the presence of PY416 SFK in the Panx1 complex. In the sham group, the relative intensity of the PY416 SFK band in the Panx1 complex was 0.69 ± 0.05 (*n* = 5, Figure 3B). A marked increase in the intensity of the PY416 SFK band to 2.3 ± 0.3 was observed in the CSD group (*n* = 6, Figure 3B), which was significantly different from that of the sham group (*p* = 0.004), suggesting that CSD promotes the interaction between activated SFK and Panx1 in the ipsilateral cortices of rats. It was noted that the preliminary study demonstrated that the total Panx1 level was not altered after CSD (*n* = 3, Figure 3C,D). These suggest that the increase of PY416 SFK observed after CSD is highly likely to attribute to the enhanced interaction of PY416 SFK with Panx1 but not the expression of Panx1 protein. 

### 2.4. NR2A Antagonism Reduced CSD-Induced Ipsilateral Cortical SFK and Panx1 Interaction and PI Staining

In order to explore if CSD-induced Panx1 activity could be regulated by NR2A-containing receptors, we investigated whether the NR2A inhibition could reduce the interaction of activated SFK and Panx1 and neuronal PI staining induced by CSD in rats. 

As seen in Figure 3B, inhibition of NR2A activity by NVP reduced the elevated level of phosphorylated SFK level from the Panx1 complex induced by CSD in ipsilateral cortices of rats (*n* = 4). The relative intensity of the phosphorylated SFK band was 1.3 ± 0.2 after NVP treatment, and this was significantly different from that of the CSD group (*p* = 0.009, Figure 3B).

**Figure 4:**
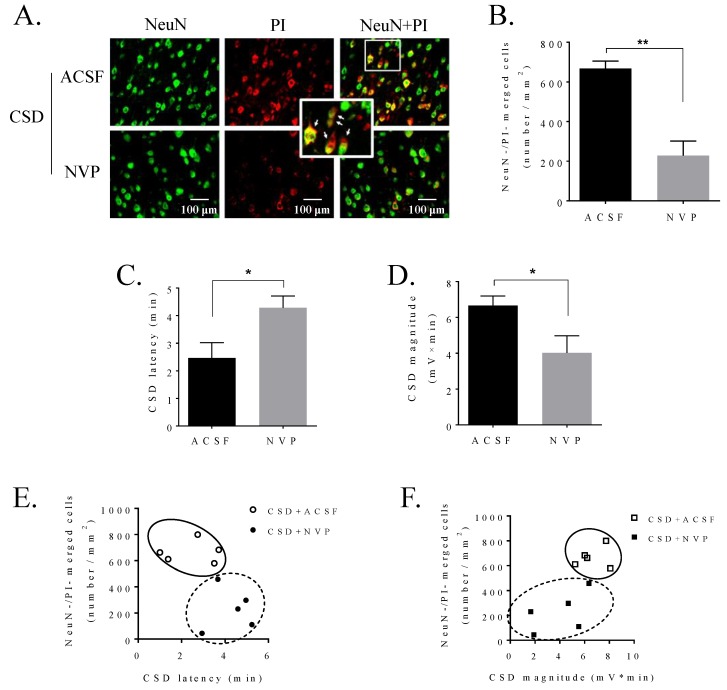
NVP, the NR2A antagonist, perfused into the contralateral i.c.v. attenuated CSD-induced neuronal PI staining in the ipsilateral cortex of rat and reduced cortical susceptibility to CSD. (**A**) Representative images of CSD-induced PI staining (red) on NeuN positive cells (green) in layers V and VI of the sensorimotor cortices pretreated with ACSF or NVP. PI and NeuN positive cells (yellow) were pointed by arrows in the inset. (**B**) Neuronal PI staining was indicated by the number of merged cells (cells/mm^2^) after pretreatment of ACSF (*n* = 5) or 0.3 nmol NVP (*n* = 5) and CSD induction in sensorimotor cortices of rats. (**C**,**D**) Effects of 0.3 nmol NVP on CSD latency (minute) and magnitude (mV × minute) (*n* = 5 for each group). (**E**,**F**) Correlation between the level of PI staining and CSD latency/CSD magnitude pretreated with ACSF or 0.3 nmol NVP (*n* = 5 for each). One-tailed, Mann–Whitney test was used for comparison between ACSF and NVP groups. All values are shown as means ± SEM. Significant differences were set when * *p* < 0.05 and ** *p* < 0.01.

We next investigated whether NR2A may regulate the neuronal PI influx induced by CSD in the ipsilateral cortex of rat. In the control group, the level of CSD-induced PI-positive staining was 667.3 ± 37.9 per mm^2^ in sensorimotor cortices of rats (*n* = 5, Figure 4A,B). Similar to the effect of PP2 on reducing PI staining (Figure 1C), i.c.v. administration of 0.3 nmol NVP also significantly reduced the number of PI-positive neurons to 228.8 ± 72.7 per mm^2^ (*n* = 5, *p* = 0.004, Figure 4A,B) when compared with that of the ACSF control group. Complementing this, NVP prolonged CSD latency (2.47 ± 0.55 min in ACSF group vs 4.28 ± 0.43 min in NVP group, *n* = 5, *p* = 0.028, Figure 4C) and reduced CSD magnitude (6.66 ± 0.54 mV × minute in ACSF group vs 4.03 ± 0.95 mV × minute in NVP group, *n* = 5, *p* = 0.048, Figure 4D), as we reported previously [20].

We then analyzed whether the reduction of CSD-induced PI staining by NR2A inhibitor was associated with cortical susceptibility to CSD. The analysis demonstrated that the lower level of PI uptake in neurons by NVP pretreatment in ipsilateral cortices of rats was tightly accompanied by the longer CSD latency and the lower CSD magnitude, suggesting that reduced cortical susceptibility to CSD might correlate with reduced PI influx by NR2A inhibition (Figure 4E,F).

## 3. Discussion

The present study reveals a novel migraine aura mechanism by which CSD induces SFK-dependent Panx1 activity, which, at least in part, is regulated by NR2A-containing receptors. 

We first find that a single CSD is capable of inducing neuronal PI staining in the ipsilateral cortex of rats (Figure 1B,C). This is consistent with a previous finding in mice that CSD promotes Panx1 opening, in which PI staining was used as an indirect measure to reflect Panx1 channel activity that is validated using Panx1 inhibitors and Panx1-small interfering RNAs [10]. It is noted that similar numbers of ipsilateral cortical PI-positive neurons in the sham group of rats (Figure 1C) and mice [10] are observed and the number of PI-positive neurons is elevated after CSD in both species; however, to a lesser extent in rats (Figure 1C) than in mice [10]. This difference may be associated with different methods used for CSD induction and the number of CSD induced in the two studies, i.e., immediate after CSD by KCl application in the present study versus the 5-min post multiple CSD by pinprick in mice [10]. Albeit PI is not a selective dye for Panx1 as the opening of large pore channels such as P2X7 receptors could also allow PI influx into neurons [25]; nevertheless, the CSD-induced PI influx in the rat cortex, at least in part, is attributed to the Panx1 channel opening. These data suggest that CSD is capable of promoting cortical Panx1 activation in the rat cortex. 

SFK is known to directly interact with the putative Y308 site at the C-terminal of Panx1 in the rat hippocampal slice model [18,19,26] and that SFK activation is required for CSD propagation [20]. Therefore, we have reasoned that activated SFK may be critical in transmitting CSD-induced Panx1 activity. Indeed, this is supported by the demonstration that SFK inhibition by PP2 perfused into contralateral i.c.v. markedly attenuates the elevated PI staining induced by CSD in the ipsilateral cortex of rats (Figure 1C). The involvement of phosphorylated SFK in CSD-induced Panx1 activity is further supported in that CSD largely promotes the interaction of phosphorylated SFK with Panx1 (Figure 3B). Complementing these findings, when the SFK-activated site of Panx1 at tyrosine 308 was interfered with by the interfering peptide, TAT-Panx308, on the mouse brain slice, a marked inhibition of cortical susceptibility to CSD was observed, as shown as the prolonged CSD latency and reduced CSD propagation rate (Figure 2). These data strongly support the action of activated SFK-dependent Panx1 activity in migraine aura pathophysiology. As the reduced cortical susceptibility to CSD is only observed by disrupting the SFK-Panx1 complex (Figure 2) but not by blockade of Panx1 channels in mice reported in a previous study [10], and TAT-Panx308 reduces Panx1 phosphorylation at Y308 [19], we propose that one model of the SFK-dependent Panx1 activity associated with CSD is potentially via direct phosphorylation of Panx1 at Y308. Further investigation is necessary to reveal how SFK interacts with Panx1 to contribute to the large-pore opening of Panx1 after CSD. Taken together, we can conclude that SFK activation is capable of regulating Panx1 activity induced by CSD via coupling of activated SFK to Panx1, which in turn affects CSD propagation. These data suggest a key role of phosphorylated SFK-dependent Panx1 activation in migraine aura pathogenesis, by which the CSD-induced neuroinflammatory response is initiated via Panx1 channels [10]. 

One possible molecular mechanism underpinning SFK-dependent Panx1 activity after CSD is its association with NMDA receptors. Firstly, SFK–Panx1 can form a complex with multiple membrane proteins including NMDAR [19], the latter of which is known to play a key role in regulating susceptibility to CSD [22]. Secondly, in the rat hippocampus slice, NMDAR–Src–Panx1 is found to co-localize in a metabotropic signaling complex [19] and NMDAR couples SFK to Panx1 during ischemia in response to glutamate and glycine but not Ca^2+^ influx through NMDAR [19]. Thirdly, NR2A-containing NMDAR functionally interacts with SFK in regulating cortical susceptibility to CSD in mouse brain slices [20] and NR2A inhibition reduces CSD-induced SFK phosphorylation in rats [20]. In our study, we further demonstrate that NR2A inhibition not only attenuates phosphorylated SFK coupling to Panx1 (Figure 3A,B), but also reduces CSD-induced neuronal PI staining in the ipsilateral cortex of rat (Figure 4A,B). The latter is consistent with the previous finding in mice that blockade of the NMDA receptors by MK801 reduces the Panx1 opening induced by CSD [10]. Notably, the reduced cortical susceptibility to CSD under NR2A inhibition (Figure 4C,D) correlates with a lower level of PI staining (Figure 4E,F). Taken together and given that the interfering peptide TAT–Panx308 is specific for NMDAR–SFK–Panx1 complex but not the SFK activity alone outside of the complex [19], we propose that CSD-induced SFK-dependent Panx1 activity is regulated by NR2A via a model in which SFK phosphorylates Panx1 at Y308, being coupled to Panx1 via NR2A.

Alternative mechanisms underlying SFK-dependent Panx1 activity induced by CSD are possible as Panx1 not only interacts with NMDAR in neurons but also with other membrane proteins such as P2X7 receptors in glial cells [27]. Since the blockade of P2X7 receptors–Panx1 pore is known to suppress CSD in vivo [17] and the tyrosine kinase signal transduction pathway mediating Panx1 activation is through P2X7 receptors, it is possible that SFK-dependent Panx1 activity induced by CSD may be also regulated by P2X7 receptors. Collectively, we propose that SFK-dependent Panx1 activity induced by CSD may be a convergent point of multiple signals including both NMDA receptor and P2X7 receptors.

It is noticeable that some Panx1 channels still remain activated even when SFK or NR2A activity is inhibited (Figure 1C and Figure 4B). The reason to account for this is unclear. One possible explanation is that the baseline PI positivity is due to the insertion of the electrode into the cortex, which may trigger a CSD. This may also be partly related to the activity of NR2B subunit of NMDAR for the following reasons: (i) Similar to NR2A, NR2B also contributes to cortical susceptibility to CSD in rats [23,28]; and (ii) NR2A and NR2B have synergistic effects on CSD propagation as co-application of NR2A and NR2B antagonists further suppresses CSD in vitro compared to single application of NR2A antagonist [29]. Given that the Ca^2+^ influx dependent-manner of NMDAR contributes to the CSD-induced Panx1 opening in mice [10], further study on whether SFK coupling to Panx1 during CSD is regulated by both NR2B and Ca^2+^ influx through NMDAR may be required. 

**Figure 5:**
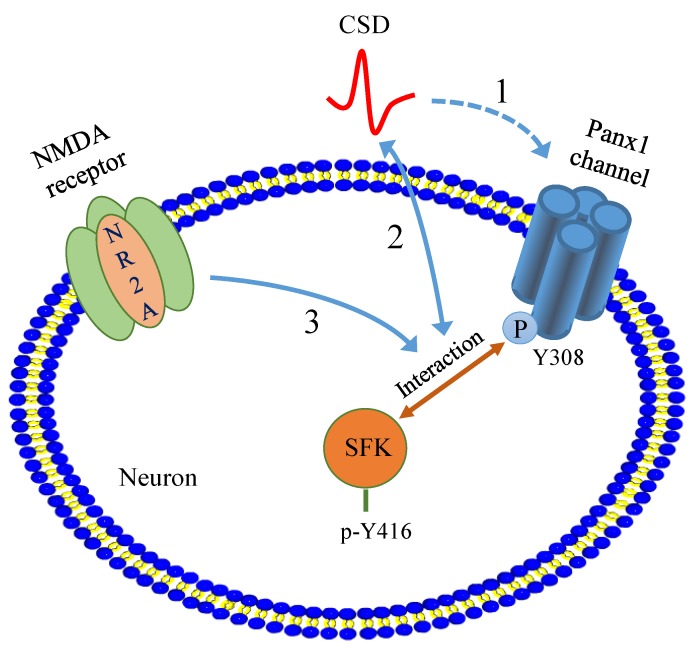
Depiction of the proposed novel migraine aura mechanism by which phosphorylated SFK is involved in transmitting CSD-induced Panx1 activation, which is regulated by NR2A. (1) CSD (red solid curve) promotes Panx1 channel activity (blue dotted line with one-sided arrow) [10], which can be facilitated by SFK activation and regulated by NR2A-containing NMDA receptors. (2) The interaction between activated SFK and Panx1 is enhanced after CSD and disruption of this interaction suppresses cortical susceptibility to CSD (blue solid line with two-sided arrows). (3) NR2A is able to mediate the coupling of SFKs to Panx1 and Panx1 activity promoted by CSD (blue solid line with one-sided arrow). Abbreviations: P refers to phosphorylation; SFK, sarcoma family kinases; Panx1, pannexin-1; CSD, cortical spreading depression; Y, tyrosine residue.

In summary, our present study reveals a novel migraine aura mechanism by which CSD induces SFK-dependent Panx1 channel activation via NR2A. Given the important role of NR2A and SFK in CSD propagation and in Panx1 activation leading to neuro-inflammatory responses, we propose that NR2A/SFK/Panx1 signaling may play an important role in migraine aura pathogenesis (Figure 5) and drugs that target this pathway might constitute an effective strategy for migraine prophylaxis. 

## 4. Materials and Methods 

### 4.1. Animals

A total of 44 adult male Sprague–Dawley rats (322.9 ± 5.8 g, mean ± SEM) and 19 adult male C57BL/6J mice (22.1 ± 0.4 g, mean ± SEM) were purchased from Shanghai SLAC Laboratory Animal Corporation Ltd. and were housed with food and water available ad libitum. All animals were housed two per cage in specific pathogen-free conditions and were allowed to acclimate to the housing room for at least 1 week before use. Animal procedures were approved by the Ethical Review Panels of Soochow University (SBK201220546) under agreement with XJTLU (Jul 2011 & Mar 2015) and performed during the light phase of the cycle in accordance with relevant national and provincial guidelines. 

### 4.2. In Vivo Experiment

#### 4.2.1. Animal Surgery and CSD Induction

Rats were anesthetized with isoflurane (5% for induction, 2.5–3.5% during surgery, 1–1.5% for maintenance) in O_2_:N_2_O (1:2), with the animal breathing spontaneously, as previously reported [24]. During experiments, the depth of anesthesia was monitored and adjusted by the absence of whisker movements, lack of reaction to brief tail pinches and thorough examination of electroencephalogram (EEG) signals. The rectal temperature of animals was maintained at 37 °C. 

Three burr holes were drilled in the parietal bone (Figure 1A, upper). One of these burr holes (coordinate: 0.8 mm posterior and 1.8 mm lateral to bregma) was drilled in the left side, which was used for implanting a stainless-steel cannula (RWD Life Science, Shenzhen, China) into the cerebral ventricle (i.c.v., 3.5 mm deep from the cortical surface) for drug perfusion. The other two burr holes were drilled in the right side: the posterior hole (coordinate: 5 mm posterior and 2 mm lateral to bregma) with dura intact was used for CSD induction; the anterior hole (coordinate: 3 mm anterior and 2 mm lateral to bregma) was drilled for Ag/AgCl electrode (0.1 mm, Applied Neuroscience, London, UK) implantation. The DC potential was derived between the Ag/AgCl electrodes and a grounded reference electrode placed under the scalp for quantifying CSD waves. The subsequent experimental procedure was carried out after at least one hour of stabilization post-surgery.

As described previously [24], CSD was induced by a careful topical application of 1 µl of 3 M KCl (Sigma-Aldrich, St. Louis, MO, USA) for 5 min. Albeit this concentration was high, it was applied for consistency with our previous publications for CSD induction [24] and this 5-min application of KCl typically elicited one CSD wave. The cortex was washed with artificial cerebrospinal fluid (ACSF, composition in mM: 2.5 NaCl, 250 KCl, 1.18 MgCl_2_, 1.26 CaCl_2_; pH 7.3 adjusted with 1 M NaOH). Animals were then immediately sacrificed as soon as CSD recordings were completed for tissue dissection or perfusion.

#### 4.2.2. Recording of EEG and Extracellular DC Potential

As reported previously [24], EEG and DC signals were amplified using an AC/DC pre-amplifier (NL834, Digitimer Ltd., Welwyn Garden City, UK). The alternating current component in 1–30 Hz provided the EEG signal (×5000 overall amplification). The DC component in 0–30 Hz provided the DC potential (×250 overall amplification). All the recorded variables were continuously displayed and recorded by Labview 11.0 (National Instruments, Austin, TX, USA) during the experiment. The spreading depolarization wave of CSD was recognized by a transient, negative shift in the DC potential, demonstrating successful CSD induction (Figure 1A, lower).

#### 4.2.3. In Vivo Experimental Design

*Series 1:* Our previous study demonstrated that deactivation of SFK by 3-(4-chlorophenyl) 1-(1,1-dimethylethyl)-1H-pyrazolo[3,4-dpyrimidin-4-amine (PP2), perfused through contralateral i.c.v. reduces CSD in rats [20]. In this study, we investigated whether SFK deactivation could suppress CSD-induced Panx1 activity in the rat ipsilateral cortex, which was associated with the reduced cortical susceptibility to CSD. Four groups were designed: (i) 2.5 nmol SFK selective inhibitor, PP2 (1407, Tocris, Bristol, UK); (ii) 2.5 nmol inactive analog of PP2, 1-Phenyl-1Hpyrazolo [3,4-d]pyrimidin-4-amine (PP3, 2794, Tocris, Bristol, UK). PP2 or PP3 was perfused into the i.c.v. at 0.5 μL/minute for 10 min using a microsyringe pump (CMA100, CMA/Microdialysis, Solna, Sweden), starting at 120 min before KCl application to allow drugs entering into the cell sufficiently; (iii) ACSF perfusion instead of PP2/PP3 in the CSD group; and (iv) sham group in the absence of KCl application. As a membrane-impermeable fluoroprobe, PI signal examined with a fluorescence microscope at 535 nm was used as an indirect measure to reflect Panx1 channel activity induced by CSD [10], then 2 μL PI (P4170, Sigma-Aldrich, St. Louis, MO, USA, 1 mg/mL) was perfused into the i.c.v. 10 min before CSD induction for PI staining.

Series 2: We next examined whether CSD could promote the interaction of activated SFK and Panx1, and if so, whether NR2A inhibition could suppress their coupling induced by CSD. Three groups were designed: (i) ACSF in the sham; (ii) ACSF in the CSD group; and (iii) 0.3 nmol the NR2A-containing receptor antagonist, NVP-AAM077 (NVP, synthesized at XJTLU), which is known to reduce cortical susceptibility to CSD [24]. Homogenates from rat cerebral cortices in the CSD group and the sham group, respectively, were also used for studying whether CSD could alter Panx1 expression. The experimental protocol was the same as that in Series 1 except that NVP, instead of PP2/PP3, was applied starting from 40 min before CSD induction.

Series 3: In order to examine whether CSD-induced PI influx could be attenuated by NR2A inhibition, two groups were designed: (i) 0.3 nmol NVP, and (ii) ACSF in the presence of KCl application. The protocol for drug perfusion was kept the same as that in Series 2.

In the above three series, only a single dose of the NR2A-containing receptor antagonist or the SFK inhibitor was used as the concentrations applied; these drugs have been previously reported to reduce cortical susceptibility to CSD [24,30].

#### 4.2.4. Histofluorescence

Immediately after CSD recording, rats from Series 1 and Series 2 were transcardially perfused with phosphate buffer saline (PBS, 09-8912-100, Medicago, Uppsala, Sweden), followed by 4% paraformaldehyde (PFA, P804537, Macklin, Shanghai, China). Brain was quickly removed, post-fixed in 4% PFA overnight and cryoprotected in 30% sucrose. Coronal sections (20 μm) from coordinates close to 0.4 mm anterior to bregma where the motor, somatosensory, and cingulate cortices were prepared by a cryostat (CM1950, Leica, Wetzlar, Germany) and stored in −80 °C. PI signal was examined with a fluorescence microscope (Eclipse Ni-U, Nikon, Tokyo, Japan) at 535 nm [10].

Brain slices were incubated with NeuN monoclonal antibody (anti-mouse, MAB377, Millipore, Temecula, CA, USA) in order to label neurons [30]. Briefly, slices were blocked with 5% goat serum (AR0009, Boster Biological Technology, Pleasanton, CA, USA) for 1 h to reduce non-specific binding. It was then exposed to the NeuN antibody at 1:1000 overnight at 4 °C, followed by incubating with a fluorescently-labeled (488) secondary antibody (A11029, Invitrogen, Carlsbad, CA, USA) at 1:500 for 1 h at room temperature (RT). Immuno-negative control was performed by staining with NeuN antibody in rat Purkinje cells [30] (data not shown).

#### 4.2.5. Protein Preparation

Cortical tissues were homogenized in lysis buffer with the presence of protease inhibitor (04693116001, Roche, Indianapolis, IN, USA) and phosphatase inhibitor (5870, CST, Beverly, MA, USA) for subsequent detection of the Panx1 expression and the interaction of Panx1 with activated SFK. Total protein was harvested from the supernatant of tissue lysis after centrifugation at 13,000 rpm for 10 min at 4 °C. Total protein concentration was determined using a bicinchoninic acid protein assay kit (P0010, Beyotime, Shanghai, China).

#### 4.2.6. Co-Immunoprecipitation

Co-immunoprecipitation was used to detect the interaction between phosphorylated SFK at Y416 (PY416 SFK) and Panx1 by using a Pierce Classic IP Kit (26146, Thermo Fisher Scientific, Waltham, MA, USA), followed by Western blotting. Briefly, 300 μg protein was pre-cleaned, followed by incubation with 100 μL buffer of 0.02 μg Panx1 antibody (LS-C138631, LSBio, Seattle, WA, USA) and 10 μL A/G protein agarose resin for 2 h at 4 °C. The bound resin was separated from the mixture at 1000× *g* for 1 min and washed in IP lysis/wash buffer following centrifugation at 1000× *g* for 1 min for three times prior to Western blotting.

#### 4.2.7. Western Blotting

Protein samples were denatured in 4× NuPAGE^®^ LDS Sample Buffer (NP0007, Invitrogen, Carlsbad, CA, USA) by boiling at 100 °C for 5 min, followed by separating on a 10% sodium dodecyl sulfate-polyacrylamide gel and transferring onto nitrocellulose membranes. Non-specific binding was blocked with 5% skim milk in tris-buffered saline with Tween-20 (TBST) for 1 h at RT.

Nitrocellulose membrane was then incubated with either PY416 SFK monoclonal antibody (anti-rabbit, 6943, CST, Beverly, MA, USA) at 1:500 or the Panx1 monoclonal antibody at 1:30000 overnight at 4 °C. Once excess primary antibody was washed off by TBST, membranes were incubated with horseradish peroxidase-labeled secondary antibody at 1:5000 for 1 h at RT. Protein bands were detected with Western bright enhanced chemiluminescence working solution (K-12045-D50, Advansta, Menlo Park, CA, USA). β-actin was detected as the reference for total Panx1 detection after stripping with the Panx1 nitrocellulose membrane using 0.2 M NaOH for 15 min at 37 °C. The same process was repeated starting from non-specific binding blocking except that β-actin monoclonal antibody (anti-rabbit, 4970, CST, Beverly, MA, USA) at 1:1000.

### 4.3. In Vitro Experiment

#### 4.3.1. Mouse Brain Slice Preparation

C57BL/6J male mice were sacrificed by cervical dislocation and brain coronal sections (400 μm) were obtained using a vibratome (7000 smz-2, Campden Instruments, Oxford, UK). The coordinates of the selected brain slices were between 1 to 3 mm posterior to bregma, which contain the somatosensory or visual cortex. The brain was incubated in oxygenated NMDG-HEPES cutting solution composed of mM: 93 NMDG, 2.5 KCl, 1.2 NaH_2_PO_4_, 30 NaHCO_3_, 20 HEPES, 25 glucose, 5 L-ascorbic acid, 2 thiourea, 3 sodium pyruvate, 10 MgSO_4_, 0.5 CaCl_2_•2H_2_O; pH 7.35–7.45) during sectioning to maintain tissue osmolality and activity, It was subsequently transferred to oxygenated Kreb’s solution (composition in mM: 126 NaCl, 2.5 KCl, 2.4 CaCl_2_•2H_2_O, 1.3 MgCl_2_•6H_2_O, 18 NaHCO_3_; 1.2 NaH_2_PO_4_, 10 glucose, pH 7.35–7.45) for minimum 80 min prior to experimentation.

#### 4.3.2. CSD Induction and Recording by Intrinsic Optical Imaging

The CSD mouse brain slice model was used as previously reported [31]. Briefly, the brain slice was placed in a chamber and submerged in Kreb’s solution with a perfusion rate of 3 mL/minute. Unless otherwise stated, CSD was induced on the somatosensory cortex of the slice by 33 μL 260 mM KCl with a syringe pump (CMA/400, CMA/ Microdialysis, Solna, Sweden). The coronal slice was illuminated for 50 ms, starting when CSD was elicited, using an LED spotlight (625 nm peak wavelength, SLS-0307-A, Mightex, Pleasanton, CA, USA) driven by an LED controller (SLC-SA04-US, Mightex, Pleasanton, CA, USA). The monochrome camera (Rolera-XR, ROL-XR-F-M-12, Qimaging, Media Cybernetics, Marlow, UK) and the LED illumination were synchronized at 2 Hz using an external trigger (TG1006, TTI, Cambridgeshire, UK). The intrinsic optical signal (IOS) in each cortical slice was recorded using Image Pro Plus (IPP7.0, Media Cybernetics, Shanghai, China) software for 15 min after KCl application in order to obtain a full CSD curve. An area of interest (AOI) distant from where KCl was applied was selected between Layers 3 to 5 of the somatosensory cortex in the merged images captured. An IOS curve was generated by plotting the average of the grey level change of pixels in the AOI against time (i.e., 1800 data points over 15 min), representing the degree of molecular changes brought by CSD.

#### 4.3.3. In Vitro Experimental Design

*Series 4*: Using the mouse brain slice, we investigated whether interruption of SFK–Panx1 interaction could alter cortical susceptibility to CSD. An interfering peptide [18], TAT–Panx308 (sequence: YGRKKRRQRRRLKVYEILPTFDVLH, A^+^ Peptide, Shanghai, China) and its scrambled control TAT–PanxSC (YGRKKRRQRRRVILLKDHTLEYPVF, A^+^ Peptide, Shanghai, China) were applied, respectively. TAT–Panx308 is a TAT-conjugated peptide that mimics the 305 to 318 amino acids of the C-terminus of Panx1, which contains part of an SFK consensus sequence (YEEI). This sequence of Panx1 contains a tyrosine 308 site, which is a putative phosphorylation site by SFK. This peptide has been shown to block Panx1 activation in anoxia [18] and phosphorylation at Y308 to effectively dissociate activated SFK from the NMDAR–Panx1 complex during neurotoxicity [19]. Three groups were designed: (i) Kreb’s control; (ii) TAT–Panx308 at 5 μM; (iii) TAT–PanxSC at 5 μM. Two or three brain slices of each mouse were used for different experimental groups in order to minimize the number of mice used. In each experiment, two CSD episodes were elicited on the mouse brain slice with a 45-min interval. In each experimental group, the respective drug was perfused into the tissue chamber 45 min before the second CSD induction.

### 4.4. Data Presentation and Statistical Analysis

The in vivo electrophysiological data, i.e., CSD latency and magnitude, were quantified as described previously [24]. Labview program was used to determine the area under the curve (AUC, mV × minute) of CSD waves to reflect CSD magnitude. Although occasionally, in the case of more than one CSD wave being elicited by the 5-min KCl perfusion, the magnitude of the first CSD wave recorded in each rat was averaged for data comparison.

For the image analysis, intrinsic optical imaging of CSD was quantified, as reported previously [30], for each CSD wave. Latency was calculated by the time interval between the starting point of KCl ejection and that of CSD elicitation. In each image sequence related to a given CSD within the AOI, the distance between two images in the same CSD wave, divided by the difference in their exposure time, allowed the calculation of CSD propagation rate (mm/minute).

The number of immunostaining cells per mm^2^ was averaged from 3 random fields of ipsilateral sensorimotor cortices between Layers V and VI at 40× objective magnification. These layers were chosen as a previous study demonstrated a preferential distribution of PI staining in these layers [32].

Protein expression analysis using Western blot was quantified using ImageJ software and data were normalized to either β-actin (for detecting Panx1 expression) or Panx1 (for detecting PY416 SFK–Panx1 interaction) for comparison.

Abnormal distribution using the D’Agostino–Pearson omnibus normality test was confirmed using Prism software. For these data, the Kruskal–Wallis test for comparison among multiple groups was used and was followed by the Mann–Whitney test for comparisons between 2 independent groups. The data were presented as mean ± SEM. Significant differences were set when * *p* < 0.05 and ** *p* < 0.01.

## References

[1] Burch R.C., Buse D.C., Lipton R.B. (2019). Migraine: Epidemiology, Burden, and Comorbidity. Neurol. Clin..

[2] Hadjikhani N., Sanchez Del Rio M., Wu O., Schwartz D., Bakker D., Fischl B., Kwong K.K., Cutrer F.M., Rosen B.R., Tootell R.B. (2001). Mechanisms of migraine aura revealed by functional MRI in human visual cortex. Proc. Natl. Acad. Sci. USA.

[3] Somjen G.G. (2005). Aristides Leao’s discovery of cortical spreading depression. J. Neurophysiol..

[4] Seidel J.L., Escartin C., Ayata C., Bonvento G., Shuttleworth C.W. (2016). Multifaceted roles for astrocytes in spreading depolarization: A target for limiting spreading depolarization in acute brain injury?. Glia.

[5] Charles A., Brennan K. (2009). Cortical spreading depression-new insights and persistent questions. Cephalalgia.

[6] Pietrobon D., Moskowitz M.A. (2013). Pathophysiology of migraine. Annu. Rev. Physiol..

[7] Russo A.F. (2015). Calcitonin Gene-Related Peptide (CGRP): A New Target for Migraine. Annu. Rev. Pharmacol. Toxicol..

[8] Zhang X., Levy D., Noseda R., Kainz V., Jakubowski M., Burstein R. (2010). Activation of meningeal nociceptors by cortical spreading depression: Implications for migraine with aura. J. Neurosci..

[9] Zhang X., Levy D., Kainz V., Noseda R., Jakubowski M., Burstein R. (2011). Activation of central trigeminovascular neurons by cortical spreading depression. Ann. Neurol..

[10] Karatas H., Erdener S.E., Gursoy-Ozdemir Y., Lule S., Eren-Kocak E., Sen Z.D., Dalkara T. (2013). Spreading depression triggers headache by activating neuronal Panx1 channels. Science.

[11] Dahl G., Locovei S. (2006). Pannexin: To gap or not to gap, is that a question?. IUBMB Life.

[12] Thompson R.J., Jackson M.F., Olah M.E., Rungta R.L., Hines D.J., Beazely M.A., MacDonald J.F., MacVicar B.A. (2008). Activation of pannexin-1 hemichannels augments aberrant bursting in the hippocampus. Science.

[13] Locovei S., Scemes E., Qiu F., Spray D.C., Dahl G. (2007). Pannexin1 is part of the pore forming unit of the P2X(7) receptor death complex. FEBS Lett..

[14] Locovei S., Wang J., Dahl G. (2006). Activation of pannexin 1 channels by ATP through P2Y receptors and by cytoplasmic calcium. FEBS Lett..

[15] Silverman W.R., de Rivero Vaccari J.P., Locovei S., Qiu F., Carlsson S.K., Scemes E., Keane R.W., Dahl G. (2009). The pannexin 1 channel activates the inflammasome in neurons and astrocytes. J. Biol. Chem..

[16] Pelegrin P., Surprenant A. (2006). Pannexin-1 mediates large pore formation and interleukin-1beta release by the ATP-gated P2X7 receptor. EMBO J..

[17] Iglesias R., Locovei S., Roque A., Alberto A.P., Dahl G., Spray D.C., Scemes E. (2008). P2X7 receptor-Pannexin1 complex: Pharmacology and signaling. Am. J. Physiol. Cell Physiol..

[18] Weilinger N.L., Tang P.L., Thompson R.J. (2012). Anoxia-induced NMDA receptor activation opens pannexin channels via Src family kinases. J. Neurosci..

[19] Weilinger N.L., Lohman A.W., Rakai B.D., Ma E.M., Bialecki J., Maslieieva V., Rilea T., Bandet M.V., Ikuta N.T., Scott L. (2016). Metabotropic NMDA receptor signaling couples Src family kinases to pannexin-1 during excitotoxicity. Nat. Neurosci..

[20] Bu F., Wang Y., Jiang L., Ma D., Quinn J.P., Wang M. (2017). Sarcoma family kinase activity is required for cortical spreading depression. Cephalalgia.

[21] Wang X.Y., Zhou H.R., Wang S., Liu C.Y., Qin G.C., Fu Q.Q., Zhou J.Y., Chen L.X. (2018). NR2B-Tyr phosphorylation regulates synaptic plasticity in central sensitization in a chronic migraine rat model. J. Headache Pain.

[22] Marrannes R., Willems R., De Prins E., Wauquier A. (1988). Evidence for a role of the N-methyl-D-aspartate (NMDA) receptor in cortical spreading depression in the rat. Brain Res..

[23] Peeters M., Gunthorpe M.J., Strijbos P.J., Goldsmith P., Upton N., James M.F. (2007). Effects of pan- and subtype-selective N-methyl-D-aspartate receptor antagonists on cortical spreading depression in the rat: Therapeutic potential for migraine. J. Pharmacol. Exp. Ther..

[24] Bu F., Du R., Li Y., Quinn J.P., Wang M. (2016). NR2A contributes to genesis and propagation of cortical spreading depression in rats. Sci. Rep..

[25] Kovacs G., Kornyei Z., Toth K., Baranyi M., Brunner J., Neubrandt M., Denes A., Sperlagh B. (2018). Modulation of P2X7 purinergic receptor activity by extracellular Zn(2+) in cultured mouse hippocampal astroglia. Cell Calcium.

[26] Thompson R.J. (2015). Pannexin channels and ischaemia. J. Physiol..

[27] Bravo D., Maturana C.J., Pelissier T., Hernandez A., Constandil L. (2015). Interactions of pannexin 1 with NMDA and P2X7 receptors in central nervous system pathologies: Possible role on chronic pain. Pharmacol. Res..

[28] Faria L.C., Mody I. (2004). Protective effect of ifenprodil against spreading depression in the mouse entorhinal cortex. J. Neurophysiol..

[29] Jia Y., Zhou J., Bu F., Wang M. (2015). Synergistic Suppression of Cortical Spreading Depression under NR2A and NR2B Inhibition. Pharmacol. Pharm..

[30] Wolf H.K., Buslei R., Schmidt-Kastner R., Schmidt-Kastner P.K., Pietsch T., Wiestler O.D., Blumcke I. (1996). NeuN: A useful neuronal marker for diagnostic histopathology. J. Histochem. Cytochem..

[31] Jiang L., Wang Y., Xu Y., Ma D., Wang M. (2018). The transient receptor potential ankyrin type 1 plays a critical role in cortical spreading depression. Neuroscience.

[32] Zoidl G., Petrasch-Parwez E., Ray A., Meier C., Bunse S., Habbes H.W., Dahl G., Dermietzel R. (2007). Localization of the pannexin1 protein at postsynaptic sites in the cerebral cortex and hippocampus. Neuroscience.

